# Burden analysis of malignant neoplasms of bone and articular cartilage in China: Epidemiological trends and future projections based on the global burden of disease study

**DOI:** 10.1097/MD.0000000000046472

**Published:** 2025-12-19

**Authors:** Le Ma, Yuke Song, Wensheng Zhang

**Affiliations:** a Guangdong Institute of Orthopedics and Trauma of Traditional Chinese Medicine, The Third Affiliated Hospital of Guangzhou University of Chinese Medicine, Guangzhou, China; b Department of Orthopedics, The Third Affiliated Hospital, Guangzhou University of Chinese Medicine, Guangzhou, China.

**Keywords:** age-period-cohort, auto-regressive integrated moving average, epidemiological trends, Global Burden of Disease, malignant neoplasms of bone and articular cartilage

## Abstract

This study investigates the epidemiological trends of malignant neoplasms of bone and articular cartilage (MNBAC) in China from 1992 to 2021, examines their association with age-period-cohort factors, and forecasts the future epidemiological trends of MNBAC for the period 2022 to 2031. Data on the incidence, prevalence, mortality, and disability-adjusted life years associated with MNBAC were sourced from the Global Burden of Disease 2021 database. Temporal trends were analyzed through age-standardized rates (ASR) and estimated annual percentage changes to capture variations over time. An age-period-cohort analysis was performed to evaluate the impact of demographic and temporal factors. Additionally, future trends for 2022 to 2031 were forecasted using the auto-regressive integrated moving average modeling approach. From 1992 to 2021, the incidence, prevalence, mortality, and disability-adjusted life years rates of MNBAC in China showed a consistent overall increase, despite a downward trend in ASR. As of 2021, the age-standardized incidence rate was recorded at 1.4 per 100,000 (95% uncertainty intervals: 0.9–1.9), with an estimated annual percentage changes of 3.27 (95% confidence intervals: 2.57–3.98). Males exhibited markedly higher rates across all metrics compared to females. The disease burden followed a bimodal age distribution, disproportionately affecting adolescents and older adults. Projections for 2022 to 2031 indicate declines in age-standardized metrics but an absolute increase in case numbers, primarily attributed to population growth and aging demographics. This analysis underscores the growing burden of MNBAC in China, marked by higher rates in males and a bimodal age distribution affecting adolescents and the elderly. While ASR are anticipated to decline in the coming decade, the overall disease burden is likely to rise persistently. These findings provide valuable insights into the epidemiological trends and determinants of MNBAC, forming a basis for targeted interventions and future research priorities.

## 1. Introduction

Malignant neoplasms of bone and articular cartilage (MNBAC) is a rare yet highly aggressive group of malignancies, primarily encompassing osteosarcoma, chondrosarcoma, Ewing sarcoma, and chordoma.^[1,2]^ These tumors exhibit significant clinical and biological heterogeneity, often characterized by rapid progression, high metastatic potential, and poor long-term survival outcomes. Although the incidence of MNBAC is relatively low, accounting for only 0.2% of all malignancies, their high disability and mortality rates impose a substantial burden on patients’ quality of life and the socioeconomic system.^[3,4]^ Global data indicate an increasing prevalence of malignant bone tumors, with significant variations in incidence and mortality across age, sex, and tumor types. For instance, osteosarcoma exhibits the highest incidence rate, while elderly patients face significantly higher mortality rates compared to other age groups. Despite the introduction of multidisciplinary treatment approaches that have improved survival outcomes for some patients, the 10-year relative survival rate for osteosarcoma remains only 61.9%, underscoring the need for further optimization of therapeutic strategies.^[3]^ In China, MNBAC presents unique regional characteristics in terms of disease burden. According to data from the Chinese Cancer Registry, both incidence and mortality rates are higher in rural areas compared to urban regions.^[5]^ In 2015, the age-standardized incidence rate (ASIR) of primary malignant bone tumors in China was 1.35 per 100,000, and the age-standardized mortality rate (ASMR) was 0.90 per 100,000. These figures are notably higher than the rates reported in the United States during the same period, with an ASIR of 1.00 per 100,000 and an ASMR of 0.50 per 100,000. Moreover, MNBAC accounts for approximately 0.62% of all newly diagnosed cancer cases in China, compared to 0.2% in the United States, highlighting the heavier disease burden in the Chinese population.^[5,6]^ Therefore, a comprehensive evaluation of the burden of MNBAC in China is essential to understand its epidemiological characteristics better and to formulate targeted public health strategies.

This study utilized the Global Burden of Disease (GBD) 2021 database to evaluate the disease burden of MNBAC in China comprehensively. The objectives included analyzing the incidence, prevalence, mortality, and disability-adjusted life years (DALYs) rates of MNBAC and examining the distribution of disease burden across different age groups and sexes. Furthermore, the study investigated the impact of age-period-cohort (APC) effects on the burden of MNBAC and projected its epidemiological trends in China over the next decade. This research represents the first comprehensive epidemiological analysis of MNBAC in China, providing critical insights into its burden on the Chinese population.

## 2. Methods

### 2.1. Overview of GBD 2021 and disease definition

The GBD 2021 study provides comprehensive insights into the burden of 371 health conditions across 204 countries and territories, encompassing a wide array of health data related to disease incidence, prevalence, mortality, and risk factors.^[7]^ All newly identified and acquired data sources are uniquely cataloged by a dedicated team of librarians and integrated into the Global Health Data Exchange (GHDx).^[7,8]^ GHDx ensures public access to metadata for each data source included in GBD, subject to the approval of data providers. Additionally, the GHDx source tool enables users to precisely locate specific datasets used to estimate health outcomes associated with particular diseases or injuries in various contexts. Detailed documentation on the data collection methods and statistical models employed in GBD 2021 has been extensively published in peer-reviewed journals. Using the Global Health Data Exchange query tool, we analyzed data on the incidence, prevalence, mortality, and DALYs of MNBAC in China from 1992 to 2021. The definition of MNBAC is based on the codes from the International Classification of Diseases, Ninth Revision (ICD-9) and Tenth Revision (ICD-10). Specifically, the ICD-10 codes for MNBAC are C40 to C40.92, C41.0 to C41.4, and C41.8 to C41.9, while the ICD-9 codes are 170 to 170.9.^[7]^ Notably, the GBD 2021 study reports MNBAC as a single aggregated category that encompasses multiple histologic subtypes, including osteosarcoma, Ewing sarcoma, and chondrosarcoma. In this study, we used the overall estimates for the combined category and did not disaggregate outcomes by histologic subtype.

### 2.2. Statistical analysis

We implemented a comprehensive evaluation framework incorporating age-standardized rates (ASR) for incidence, prevalence, mortality, and DALYs to examine the burden of MNBAC across China. Notably, we present age-standardized incidence and mortality as rates per 100,000 person-years, prevalence per 100,000 population, and DALY rates per 100,000 person-years, each with 95% uncertainty intervals (95% UI). To address statistical uncertainty, we calculated 95% UI for each rate. Temporal trend analysis was conducted using ASR, specifically by calculating the estimated annual percentage changes (EAPC) in these metrics between 1992 and 2021. All measurements were accompanied by 95% confidence intervals (95% CI) to ensure robust statistical interpretation. The estimation of EAPC employed a linear regression model defined by the equation *y* = *a* + *bx* + *e*, where *y* represents the natural logarithm of ASR, *x* denotes the calendar year, and *b* signifies the regression coefficient. The EAPC was subsequently derived using the formula *EAPC* = 100 × (10^*b*^ − 1). In our trend analysis of ASR, we specifically examined the lower bound of the 95% CI. A positive lower bound indicated an increasing trend, while a negative value suggested a decreasing trend. Additionally, when the 95% CI contained 0, we interpreted this as a decreasing trend.

The APC model is widely used in sociology and epidemiology to analyze time trends in incidence or mortality rates based on age, period, and cohort. Built on a Poisson distribution, the APC model effectively captures these trends. The age effect reflects the influence of aging and demographic factors, such as population aging, on disease and mortality. The period effect accounts for changes in risk due to external factors, including medical advancements or policy changes, during specific periods. The cohort effect examines how varying exposures to risk factors across different birth cohorts shape health outcomes. Traditional statistical methods often face challenges in disentangling the collinearity among these 3 factors. However, the Intrinsic Estimator method overcomes this limitation, enabling the independent evaluation of age, period, and cohort effects. The APC model is defined as: *In (Refg*) = α + *Ae* + *Pf* + *Cg*, where *Refg* represents the incidence or mortality rate for the *g* birth cohort, *e* denotes the age group, and *f* refers to the period. The terms *Ae*, *Pf*, and *Cg* represent the respective effects of age, period, and cohort. In this study, we utilized the widely-recognized web-based tool developed by Philip S. Rosenberg, which has become a standard resource for APC analysis in cancer epidemiology, to conduct our APC analysis of cancer incidence and mortality trends.^[9]^

The auto-regressive integrated moving average (ARIMA) model forecasts the future trends of MNBAC incidence, prevalence, mortality, and DALYs for the next decade (2022–2031). This model requires 3 key parameters: *P*, which defines the order of the auto-regressive process to capture the impact of past values on the current value; *d*, which specifies the degree of differencing to achieve stationarity by removing trends or seasonality; and *q*, which determines the order of the moving average process to incorporate dependencies between observations and residual errors from prior steps.^[10]^ Using the auto.arima() function, we tested various combinations of *p*, *d*, and *q* to establish the best-fitting model.^[11]^ Metrics like the Akaike Information Criterion and Bayesian Information Criterion guided the selection of the optimal model by balancing accuracy and complexity, where lower values indicated a better fit.^[12]^ The final ARIMA (*p*, *d*, *q*) model enabled reliable predictions of MNBAC’s disease burden from 2022 to 2031. To ensure the model’s adequacy, we conducted the Ljung–Box *Q* test to confirm that the residuals exhibited independent and normal distributions.^[12,13]^ All statistical analyses were performed using R software (version 4.4.1; https://cran.r-project.org).

## 3. Results

### 3.1. Incidence, prevalence, mortality, and DALYs

Table 1 illustrates the epidemiological trends of MNBAC in China from 1992 to 2021, including total cases and gender-specific distributions. Over the 3-decade period, significant increases were observed in incidence, prevalence, mortality, and DALYs rates. In 2021, the ASIR of MNBAC reached 1.4 (95% UI: 0.9–1.9), with an EAPC of 3.27 (95% CI: 2.57–3.98). The most substantial increase was observed in prevalence, with an ASR of 9.2 (95% UI: 5.8–12) and an EAPC of 3.38 (95% CI: 2.68–4.08). The ASMR was 0.9 (95% UI: 0.6–1.2) with an EAPC of 2.06 (95% CI: 1.31–2.83), while the DALYs rate ASR was 29.5 (95% UI: 18.9–38.7) with an EAPC of 1.71 (95% CI: 1.01–2.43). The gender-stratified analysis revealed upward trends across all metrics for males and females, with males consistently exhibiting higher rates across all indicators. Specifically, in 2021, the ASIR for males was 1.8 (95% UI: 1–2.5), with an EAPC of 3.61 (95% CI: 2.91–4.31), which was higher than that for females, whose ASIR was 1.1 (95% UI: 0.6–1.5) and EAPC was 2.75 (95% CI: 2.03–3.47). Regarding prevalence, males had an age-standardized prevalence rates (ASPR) of 11.3 (95% UI: 6.5–15.6), with an EAPC of 3.73 (95% CI: 3.04–4.42), compared to females, whose ASPR was 7 (95% UI: 4.1–10.1) and EAPC was 2.84 (95% CI: 2.12–3.57). For mortality, males exhibited an ASMR of 1.2 (95% UI: 0.7–1.6), with an EAPC of 2.34 (95% CI: 1.58–3.1), compared to females, whose ASMR was 0.7 (95% UI: 0.4–1) and EAPC was 1.66 (95% CI: 0.9–2.43). In terms of DALYs, males had an ASR of 36.9 (95% UI: 21.4–51.3), with an EAPC of 2.05 (95% CI: 1.36–2.76), while females had an ASR of 22.2 (95% UI: 13.3–31.9) and an EAPC of 1.19 (95% CI: 0.46–1.94).

**Table 1:** ASR of MNBAC in 1992 and 2021, alongside sex-specific temporal trends from 1992 to 2021.

Measures	1992		2021		1992–2021
	Incident cases No. × 10^3^ (95% UI)	ASR per 100,000 No. (95% UI)	Incident cases No. × 10^3^ (95% UI)	ASR per 100,000 No. (95% UI)	EAPC No. (95% CI)
Incidence					
Total	6364.7 (4108.5–11606.8)	0.6 (0.4–1.1)	25937.8 (16243.1–34274.4)	1.4 (0.9–1.9)	3.27 (2.57–3.98)
Male	3669.5 (2339–6642.3)	0.7 (0.5–1.3)	15799.3 (8943.3–22305.1)	1.8 (1–2.5)	3.61 (2.91–4.31)
Female	2695.1 (1656.5–5747.7)	0.5 (0.3–1.1)	10138.5 (5954.9–14563)	1.1 (0.6–1.5)	2.75 (2.03–3.47)
Prevalence					
Total	41480.1 (26826.4–75851.5)	3.9 (2.5–7.1)	166568.7 (104517.3–219817.7)	9.2 (5.8–12)	3.38 (2.68–4.08)
Male	23838.8 (15240–43014.4)	4.5 (2.8–8.1)	100981.6 (57392.7–142644.9)	11.3 (6.5–15.6)	3.73 (3.04–4.42)
Female	17641.3 (10854.6–37734)	3.4 (2.1–7.2)	65587.1 (38468.1–94478.4)	7 (4.1–10.1)	2.84 (2.12–3.57)
Mortality					
Total	5215.5 (3301.9–9477.9)	0.6 (0.4–1)	18084.5 (11288.1–24125.6)	0.9 (0.6–1.2)	2.06 (1.31–2.83)
Male	3023.6 (1872–5525.8)	0.7 (0.4–1.2)	10833.5 (6169–15353.6)	1.2 (0.7–1.6)	2.34 (1.58–3.1)
Female	2191.9 (1343.5–4618.4)	0.5 (0.3–1)	7251.1 (4258.1–10318.9)	0.7 (0.4–1)	1.66 (0.9–2.43)
DALYs					
Total	210307.5 (134529.7–387999.9)	19.1 (12.2–35.1)	527283.6 (331726.9–699456.3)	29.5 (18.9–38.7)	1.71 (1.01–2.43)
Male	123343.9 (78419.1–221655.6)	22.2 (14–40)	324212.7 (186905.3–460067.6)	36.9 (21.4–51.3)	2.05 (1.36–2.76)
Female	86963.6 (53093.5–191160.5)	16.1 (9.9–35.1)	203070.9 (120616.2–291485.3)	22.2 (13.3–31.9)	1.19 (0.46–1.94)

ASR = age-standardized rate, CI = confidence interval, DALYs = disability-adjusted life years, EAPC = estimated annual percentage change, MNBAC = malignant neoplasms of bone and articular cartilage, UI = uncertainty interval.

**Figure 1.**
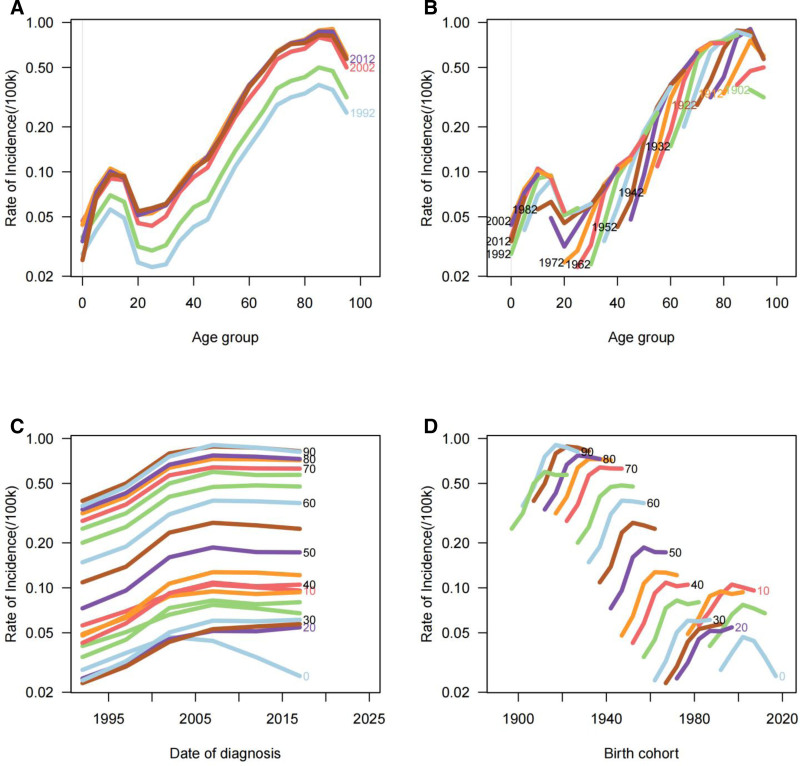
Age-specific distribution of MNBAC disease burden in China, 1992 to 2021: analysis of 4 key epidemiological measures. (A) Age-specific trends in MNBAC incidence, demonstrating the distribution of new cases across different age groups. (B) Age-specific patterns of MNBAC prevalence, showing the total disease burden among various age cohorts. (C) Age-specific mortality trends of MNBAC, illustrating death rates across age groups. (D) Age-specific distribution of MNBAC-related DALYs, representing the comprehensive measure of disease burden combining years of life lost and years lived with disability. DALYs = disability-adjusted life years, MNBAC = malignant neoplasms of bone and articular cartilage.

The age-stratified analysis from 1992 to 2021 reveals a distinct bimodal distribution across all metrics, with notable peaks observed in both younger and older populations (Fig. 1). In 2021, incidence and prevalence rates exhibit a pronounced bimodal pattern, with the highest rates in the 10 to 14 and 65 to 69 age groups. Mortality rates display a similar trend, peaking in the 15 to 19 and 70 to 74 age groups, while DALYs reach their highest levels in the 15 to 19 and 65 to 69 age groups. Notably, incidence rates show the most substantial increases in the 30 to 34 and 60 to 64 age groups, with EAPCs of 3.902 (95% CI: 3.208–4.6) and 3.997 (95% CI: 3.223–4.776), respectively. Regarding prevalence, the most dramatic increases are observed in the 90 to 94 and 95+ age groups, with EAPCs of 4.267 (95% CI: 3.441–5.099) and 4.291 (95% CI: 3.578–5.009). Mortality rates rise significantly in the 80 to 84 and 85 to 89 age groups, marked by EAPCs of 2.708 (95% CI: 1.914–3.509) and 2.649 (95% CI: 1.795–3.511), respectively. Similarly, DALYs significantly increase in these same age groups, with EAPCs of 2.7 (95% CI: 1.911–3.494) and 2.65 (95% CI: 1.808–3.499). All other age groups exhibit upward trends except for the under-5 age group, which demonstrates a declining trend across incidence, prevalence, mortality, and DALYs (Tables S1–S4, Supplemental Digital Content, https://links.lww.com/MD/Q929).

### 3.2. The effects of age, period, and cohort on incidence and mortality rates in China

**Figure 2.**
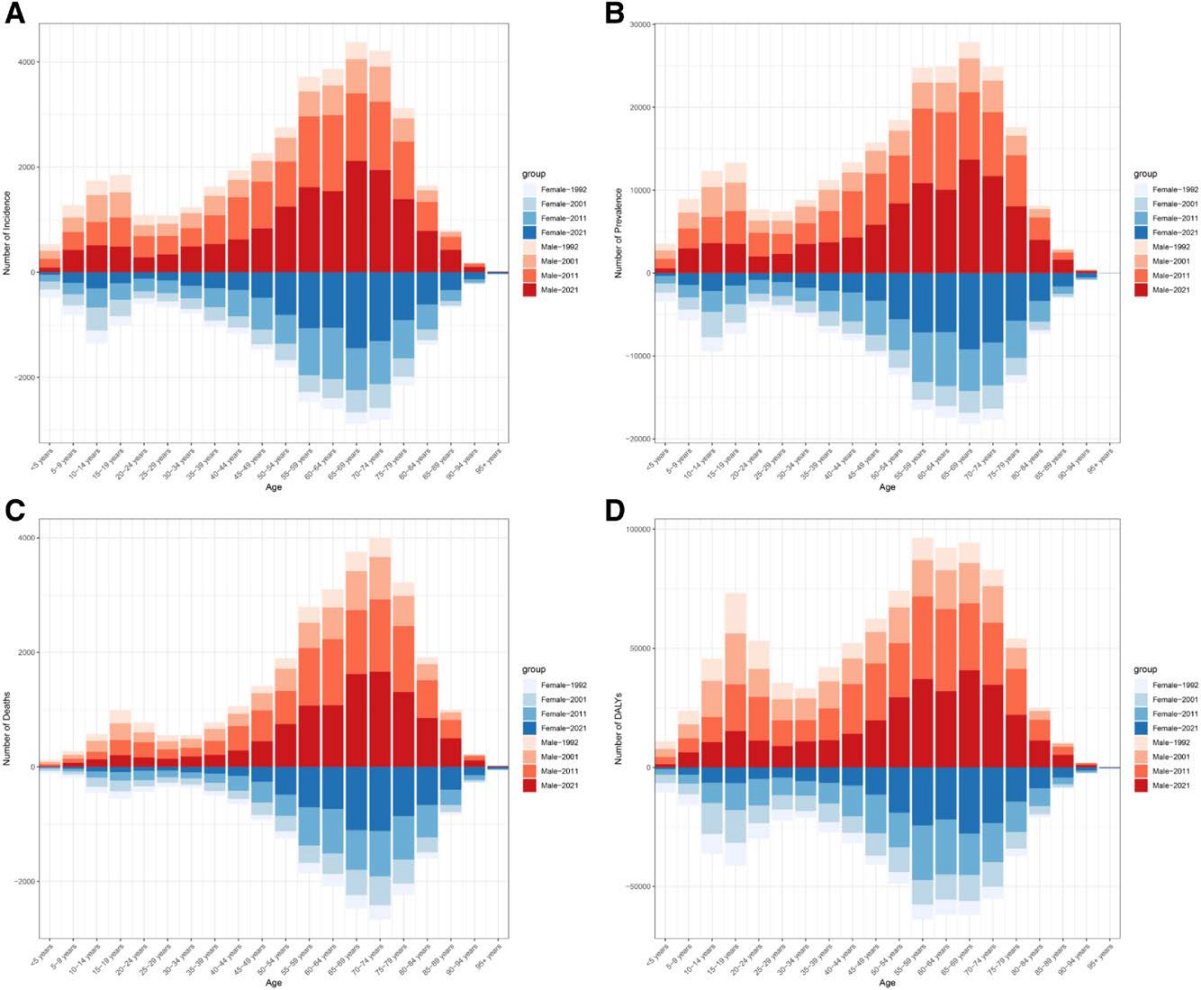
Incidence rates of MNBAC in China. (A) The age-specific incidence rates of MNBAC according to time periods; each line connects the age-specific incidence for a 5-year period. (B) The age-specific incidence rates of MNBAC according to birth cohort; each line connects the age-specific incidence for a 5-year cohort. (C) The period-specific incidence rates of MNBAC according to age groups; each line connects the birth cohort-specific incidence for a 5-year age group. (D) The birth cohort-specific incidence rates of MNBAC according to age groups; each line connects the birth cohort-specific incidence for a 5-year age group. MNBAC = malignant neoplasms of bone and articular cartilage.

**Figure 3.**
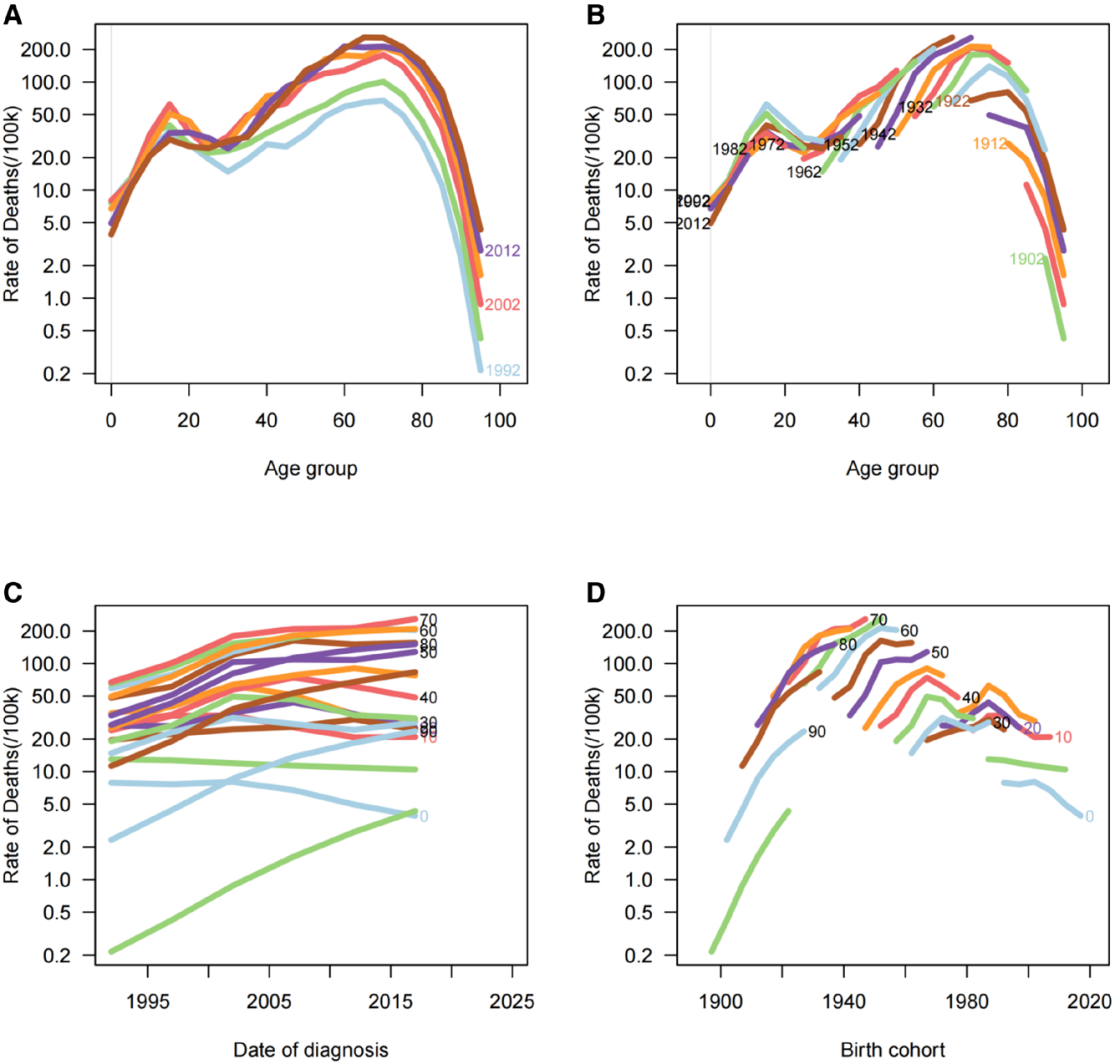
Mortality rates of MNBAC in China. (A) The age-specific mortality rates of MNBAC according to time periods; each line connects the age-specific mortality for a 5-year period. (B) The age-specific mortality rates of MNBAC according to birth cohorts; each line connects the age-specific mortality for a 5-year cohort. (C) The period-specific mortality rates of MNBAC according to age group; each line connects the birth cohort-specific mortality for a 5-year age group. (D) The birth cohort-specific mortality rates of MNBAC according to age groups; each line connects the birth cohort-specific mortality for a 5-year age group. MNBAC = malignant neoplasms of bone and articular cartilage.

Figure 2A and Figure 3A demonstrate the age-specific trends of MNBAC incidence and mortality rates in China from 1992 to 2021. The overall MNBAC incidence exhibits an upward trend characterized by a bimodal distribution, with the initial peak occurring in the 0 to 20 age group and a subsequent peak in the 60 to 80 age group. A notable progressive increase in incidence rates is observed after age 40. Similarly, MNBAC mortality rates show a gradual upward trend with a bimodal pattern, displaying a minor peak in the 0 to 20 age group and a more pronounced peak in the 60 to 80 age group, with elderly populations experiencing substantially higher mortality rates compared to younger individuals.

Figure 2B and Figure 3B elucidate the cohort trends of MNBAC incidence and mortality rates across age groups, while Figure 2C and Figure 3C depict period effects from 1992 to 2021. Period effect analysis reveals elevated incidence and mortality rates among elderly populations. Before 2005, incidence rates showed an increasing trend before stabilizing, whereas overall mortality rates demonstrated rapid growth. After 2005, the overall rising trend in mortality rates began to moderate; however, mortality rates continued to increase among elderly individuals while remaining relatively stable in younger populations.

Figure 2D and Figure 3D illustrate the birth cohort-specific trends in MNBAC incidence and mortality rates. Across all birth cohorts, overall incidence rates increase with age, showing particularly marked elevation after age 40. While mortality rates generally increase with age, they demonstrate a declining trend after age 70. Elderly populations maintain higher mortality rates, whereas younger populations show relatively stable rates. Sex-stratified analyses are presented (Figures S1–S4, Supplemental Digital Content, https://links.lww.com/MD/Q929).

After adjusting for period and cohort effects, age emerges as the principal determinant of both incidence and mortality rates. These rates display positive age-related slopes, with a particularly steep increase between ages 60 to 80, indicating accelerated disease risk, before plateauing after age 80. The age-dependency of mortality is particularly pronounced, showing gradual elevation from ages 0 to 20, followed by rapid acceleration after age 60, reaching its peak at age 80. Period effect analysis indicates temporal increases in incidence rates, particularly among elderly populations. Cohort effect analysis demonstrates higher incidence and mortality rates in earlier birth cohorts compared to recent ones, suggesting decreased risk in latter cohorts. Temporal analysis of mortality rates shows a declining trend, especially pronounced in elderly populations. The overall cohort effect pattern indicates progressive risk reduction from earlier to more recent birth cohorts.

### 3.3. Projected trends in MNBAC: incidence, prevalence, mortality, and DALYs rates 2022 to 2031

This study utilized ARIMA modeling to predict the epidemiological trends of MNBAC over the next decade, based on the GBD database from 1992 to 2021. The auto. arima() function was employed to select the optimal ARIMA model, automatically identifying the need for differencing to stabilize variance in the time series. The selection was further evaluated using the Akaike Information Criterion and Bayesian Information Criterion. The model’s robustness was validated using the Ljung–Box test, which detected no significant residuals, indicating that the selected model fits the data well.

**Figure 4.**
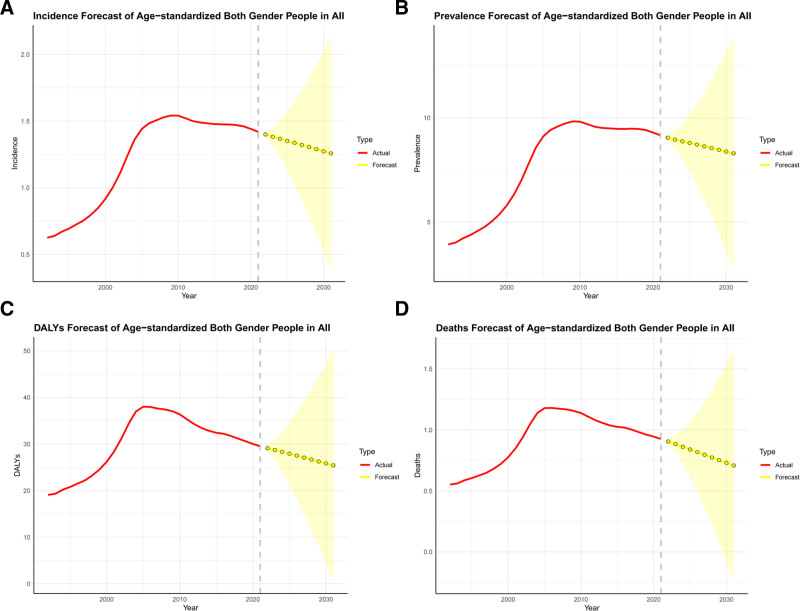
Projected trends in incidence, prevalence, DALYs, and mortality of MNBAC in China from 2022 to 2031. (A–D) The red lines representing the observed trends in age-standardized incidence, prevalence, DALYs, and mortality from 1992 to 2021, respectively, for both males and females. The yellow lines illustrate the projected trends, while the light-yellow shaded areas denote these forecasts’ 95% confidence intervals. The gray dotted vertical line marks the division between historical data (1992–2021) and projected data (2022–2031). DALYs = disability-adjusted life years, MNBAC = malignant neoplasms of bone and articular cartilage.

The projections for 2022 to 2031 reveal a declining trend in MNBAC ASR across all 4 key metrics. By 2031, these rates are estimated to be 1.26 per 100,000 for incidence, 8.29 per 100,000 for prevalence, 0.71 per 100,000 for mortality, and 25.43 per 100,000 for DALYs (Fig. 4). Predictions of incidence, prevalence, mortality, and DALYs disaggregated by gender for the same period are also included (Tables S5 and S6, Supplemental Digital Content, https://links.lww.com/MD/Q929).

## 4. Discussion

This study utilizes the GBD 2021 database to conduct the 1st comprehensive analysis of the epidemiological characteristics and trends in the burden of MNBAC in China from 1992 to 2021. Although the incidence of MNBAC remains relatively low, its high disability and mortality rates significantly affect patients’ quality of life and impose substantial burdens on the socioeconomic system. With the acceleration of population growth and aging, the burden of MNBAC in China has continued to intensify. Over the past 3 decades, the incidence, prevalence, mortality, and DALYs rates of MNBAC have shown an overall increasing trend.

The findings reveal a distinct bimodal distribution of MNBAC incidence, prevalence, mortality, and DALYs rates, with significant variations across age and gender groups. Among age groups, incidence and prevalence peak in the 0 to 14 and 65 to 69 age ranges, with the trend becoming particularly pronounced in individuals aged 60 years and older. Mortality rates exhibit a similar bimodal pattern, with peaks observed in the 15 to 19 and 70 to 74 age groups, and older adults experiencing higher mortality compared to younger populations. DALYs rates also reach their highest levels in the 15 to 19 and 65 to 69 age groups, with older individuals consistently showing higher DALYs rates than younger counterparts. Gender differences further highlight a disproportionate burden on males, who exhibit higher rates of incidence, prevalence, mortality, and DALYs compared to females. Our findings of this study align closely with previous global research on MNBAC, highlighting significant differences across age groups, particularly the high incidence and disease burden observed in adolescents and the elderly.^[5,14,15]^ For instance, global studies have demonstrated a bimodal pattern in MNBAC incidence, with the 1st peak occurring during adolescence and the second in older adulthood, which is consistent with the results of our analysis.^[15]^ Osteosarcoma and Ewing sarcoma are the most common malignant bone tumors, predominantly affecting children and adolescents, while chondrosarcoma and chordoma are more prevalent in older adults.^[3,16–18]^ Advancements in technology have led to improvements in the diagnostic and therapeutic approaches for MNBAC.^[19,20]^ Since 2005, the rising trend in MNBAC mortality in China has shown signs of stabilization, likely attributable to improved access to cancer treatment and significant gains in patient survival rates.

In recent years, the integration and expansion of urban and rural basic medical insurance have markedly enhanced access to cancer treatment across the country.^[21]^ Nevertheless, concerted efforts remain necessary to mitigate the financial burden on patients, particularly regarding expensive innovative therapies and advanced diagnostic technologies.^[22]^ Recent healthcare reforms in China have improved access to cancer treatment by expanding the inclusion of anticancer drugs in the national health insurance reimbursement list.^[23]^ Concurrently, policies promoting collaboration between public and private healthcare sectors have been established, including pilot programs that introduce commercial health insurance as a supplement to basic medical insurance.^[24]^

China’s national strategies, such as the Healthy China 2030 initiative, alongside the Cancer Prevention and Control Action Plan (2015–2017), have further prioritized strengthening cancer control and management, aiming to reduce the economic burden of treatment.^[25]^ However, despite these advancements, the overall mortality rate of MNBAC in China continues to rise. Similar to trends observed in other low and middle-income countries, financial protection for cancer care remains insufficient.^[26]^ While near-universal health insurance coverage has been achieved through substantial government investments, significant barriers persist due to high deductibles and co-payment requirements, particularly in specialized cancer centers. Additionally, many emerging diagnostic technologies and innovative therapies remain excessively costly and fall outside the scope of basic health insurance coverage.^[22,24]^ The 2020 World Health Organization Cancer Report highlights that the actual impact of many innovative diagnostic and therapeutic approaches on cancer control remains limited, emphasizing the need for further evaluations to determine their cost-effectiveness.^[22]^ Strengthening efforts to balance accessibility, affordability, and quality of cancer care will be essential in addressing the financial and clinical challenges posed by MNBAC in China.^[25,27]^

The development of MNBAC involves multiple contributing factors. Hereditary cancer syndromes, such as Li–Fraumeni syndrome and hereditary retinoblastoma, are strongly associated with germline mutations in key tumor suppressor genes (TP53 and RB1), significantly increasing the risk of osteosarcoma.^[16]^ On a molecular level, immunogenic cell death and aging-related gene signatures play critical roles in the pathogenesis and prognosis of MNBAC, potentially influencing disease progression by modulating the tumor immune microenvironment.^[28,29]^ Notably, autoimmune diseases such as rheumatoid arthritis represent independent risk factors for MNBAC, likely arising from genetic susceptibility, gene–environment interactions, and prolonged use of antirheumatic medications.^[30]^ In addition, ionizing radiation exposure and alkylating chemotherapeutic agents may induce osteosarcoma in a dose-dependent manner, serving as potential etiological factors.^[31,32]^

ARIMA projections suggest that the future burden of MNBAC will exhibit a “dual characteristic”: while individual risk, as indicated by declining ASIRs, is anticipated to decrease, the overall disease burden is expected to intensify due to an increasing number of cases. This trend is primarily driven by population aging and growth, with the continuous expansion of the elderly population playing a significant role in the rising case numbers. MNBAC incidence is disproportionately higher among older adults, and as the aging population grows, the absolute number of cases is projected to rise correspondingly. Elderly populations exhibit substantially elevated incidence and mortality rates in comparison with younger age groups, underscoring the significant correlation between aging and disease burden.^[3]^ Cohort and period effects jointly drive the projected decline in ASR. Newer birth cohorts carry lower risks (Fig. 2D and Fig. 3D). After 2005, mortality increases slowed and rates in younger groups stabilized (Fig. 3C), aligning with expanded insurance coverage, improved access to anticancer therapies, and strengthened multidisciplinary care. Under fixed standard-population weights, stable or falling age-specific rates in younger and middle-aged groups suffice to pull the overall ASR downward, even if rates in older adults remain stable or rise slightly. In parallel, declining ASIRs together with improved survival modestly lowers ASPR and DALY ASR (Fig. 4). These projections report 95% UIs; continued surveillance will be necessary to confirm the trajectory.

This study represents systematic analysis of the epidemiological trends of MNBAC in China using data from the GBD 2021 database, highlighting the characteristics of the disease burden and its potential influencing factors. Despite its strengths, this study has limitations. GBD 2021 reports national and regional estimates but lacks provincial and urban–rural detail, limiting subnational analyses. Because GBD uses standardized models to synthesize heterogeneous sources, accuracy depends on data density and context; model-related bias may vary across income settings, and rare cancers may remain under-ascertained. The database also omits key risk factors (e.g., environmental and genetic data), constraining comprehensive attribution. Cross-provincial differences in information systems, ascertainment, certification, and ICD coding introduce reporting heterogeneity that modeling cannot fully resolve. Our ARIMA projections extend observed trends and require validation in real-world settings. Future work should link provincial cancer registries to examine regional disparities and clarify how genetic, environmental, and socioeconomic factors shape MNBAC risk. Given limited histologic granularity for China in GBD 2021, we analyzed MNBAC as an aggregated category; integrating ICD-O-coded registries will enable subtype-specific analyses (osteosarcoma, Ewing sarcoma, chondrosarcoma). For high-risk groups (individuals with relevant hereditary cancer syndromes or prior high-dose therapeutic irradiation, and age bands with higher incidence (adolescents/young adults for osteogenic/Ewing tumors; older adults for chondrosarcoma)) research should develop precise screening tools and tailored interventions to optimize resource allocation and reduce burden. Real-world studies remain essential to validate ARIMA projections and support rigorous prevention strategies.

## 5. Conclusion

This study highlights the substantial disease burden of MNBAC in China. Between 1992 and 2021, males exhibited higher incidence, prevalence, mortality, and DALYs rates compared to females. The disease burden was particularly pronounced among adolescents and the elderly, demonstrating a bimodal distribution. Over the past 3 decades, the incidence, prevalence, mortality, and DALYs rates of MNBAC have shown an overall upward trend. Although ASR for these metrics are projected to decline over the next decade, the total number of cases is expected to continue increasing due to population growth and accelerated aging. This study provides valuable insights into the trends and determinants of MNBAC disease burden in China over the last 30 years, offering a scientific basis for the development of targeted prevention and health management strategies, while also identifying key directions for future research.

## Acknowledgments

We appreciate the outstanding works by the Global Burden of Diseases, Injuries, and Risk Factors Study 2021 collaborators.

## Author contributions

**Conceptualization:** Le Ma.

**Data curation:** Yuke Song, Wensheng Zhang.

**Formal analysis:** Le Ma, Yuke Song, Wensheng Zhang.

**Software:** Le Ma, Yuke Song, Wensheng Zhang.

**Visualization:** Le Ma, Yuke Song, Wensheng Zhang.

**Writing – original draft:** Le Ma.

**Writing – review & editing:** Le Ma.

## Supplementary Material


