# Supplementary material for: Burden analysis of malignant neoplasms of bone and articular cartilage in China: Epidemiological trends and future projections based on the global burden of disease study
Source: Medicine (Baltimore). 2025 Dec 19;104(51):e46472. doi: 10.1097/MD.0000000000046472 (PMC12727263; doi:10.1097/MD.0000000000046472)
Supplement: Supplementary file 1 [file medi-104-e46472-s001.pdf]

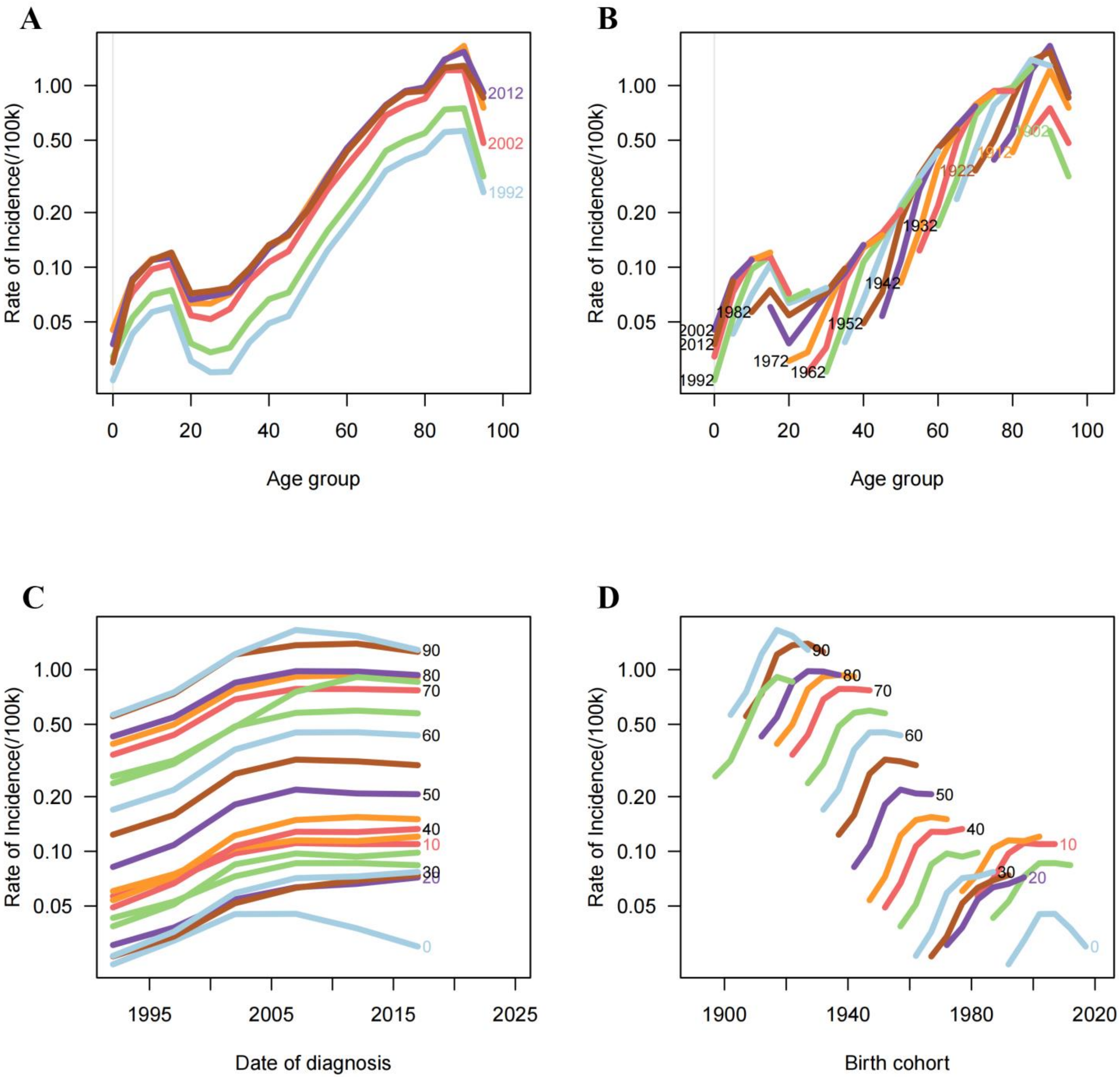
A
B
C
D
Rate of Incidence(/100k)
1.00
0.50
0.20
0.10
0.05
Age group
0
20
40
60
80
100
2012
2002
1992
1982
1972
1962
1952
1942
1932
1922
1912
1902
90
80
70
60
50
40
30
20
10
0
Date of diagnosis
1995
2005
2015
2025
Birth cohort
1900
1940
1980
2020

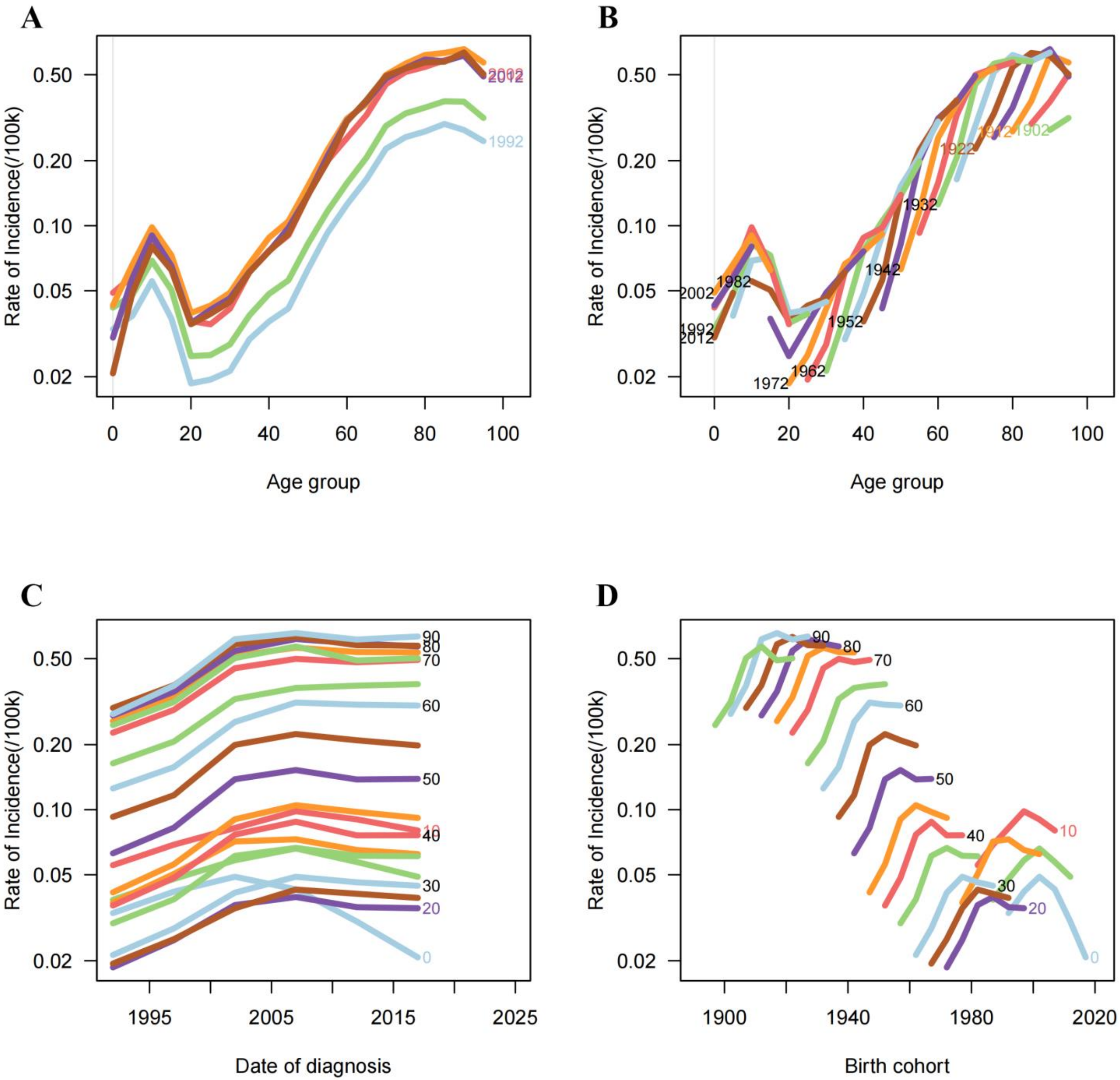
A
Rate of Incidence(/100k)
0.50
0.20
0.10
0.05
0.02
0
20
40
60
80
100
Age group
2002
2012
1992
B
Rate of Incidence(/100k)
0.50
0.20
0.10
0.05
0.02
0
20
40
60
80
100
Age group
2002
1982
1992
2012
1972
1962
1952
1942
1932
1922
1912
1902
C
Rate of Incidence(/100k)
0.50
0.20
0.10
0.05
0.02
1995
2005
2015
2025
Date of diagnosis
90
80
70
60
50
10
40
30
20
0
D
Rate of Incidence(/100k)
0.50
0.20
0.10
0.05
0.02
1900
1940
1980
2020
Birth cohort
90
80
70
60
50
40
10
30
20
0

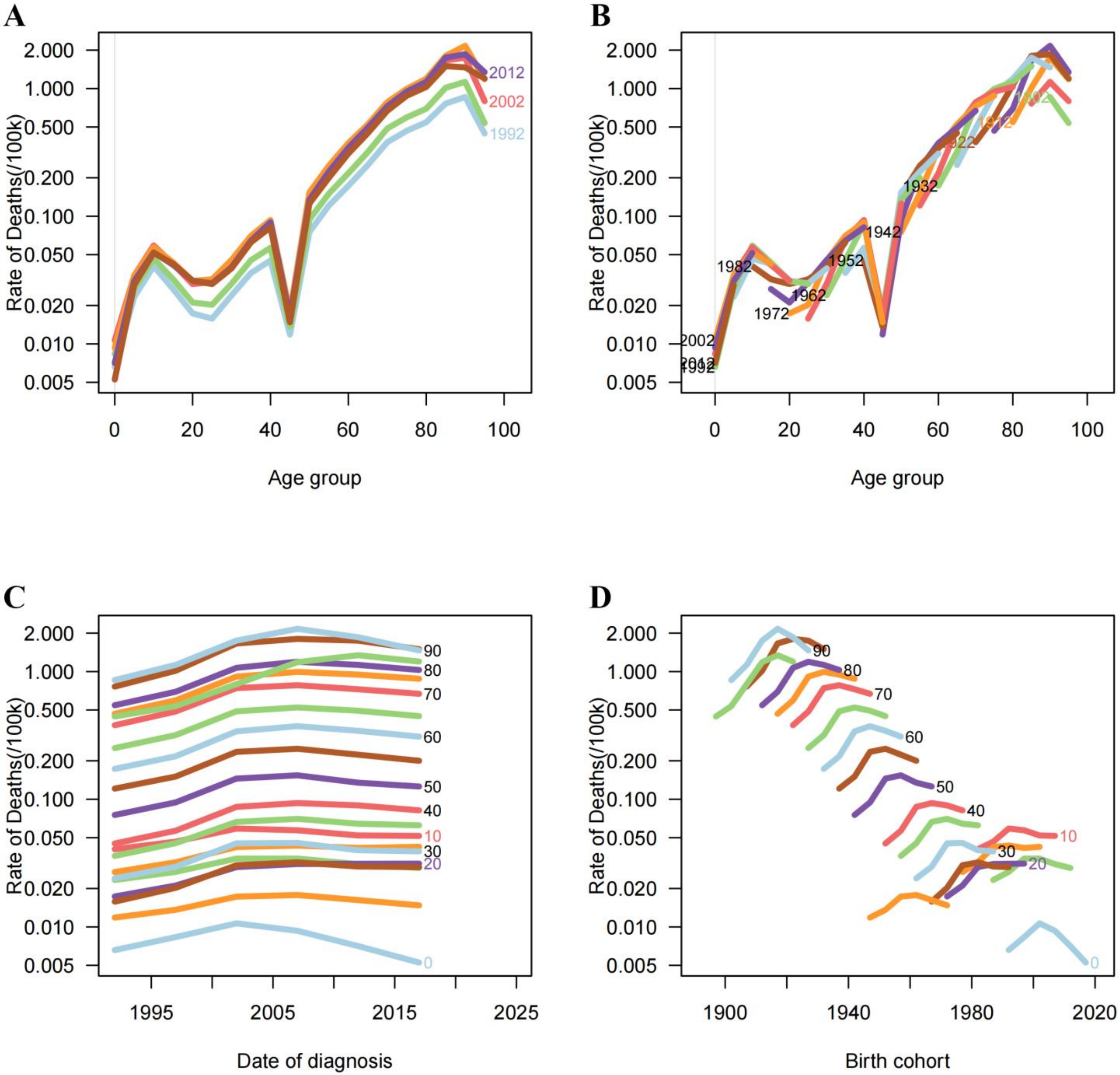
A
B
C
D
Rate of Deaths(/100k)
2.000
1.000
0.500
0.200
0.100
0.050
0.020
0.010
0.005
Age group
0
20
40
60
80
100
2012
2002
1992
1932
1942
1952
1962
1972
1982
1912
1922
Date of diagnosis
1995
2005
2015
2025
Birth cohort
1900
1940
1980
2020
90
80
70
60
50
40
10
30
20
0

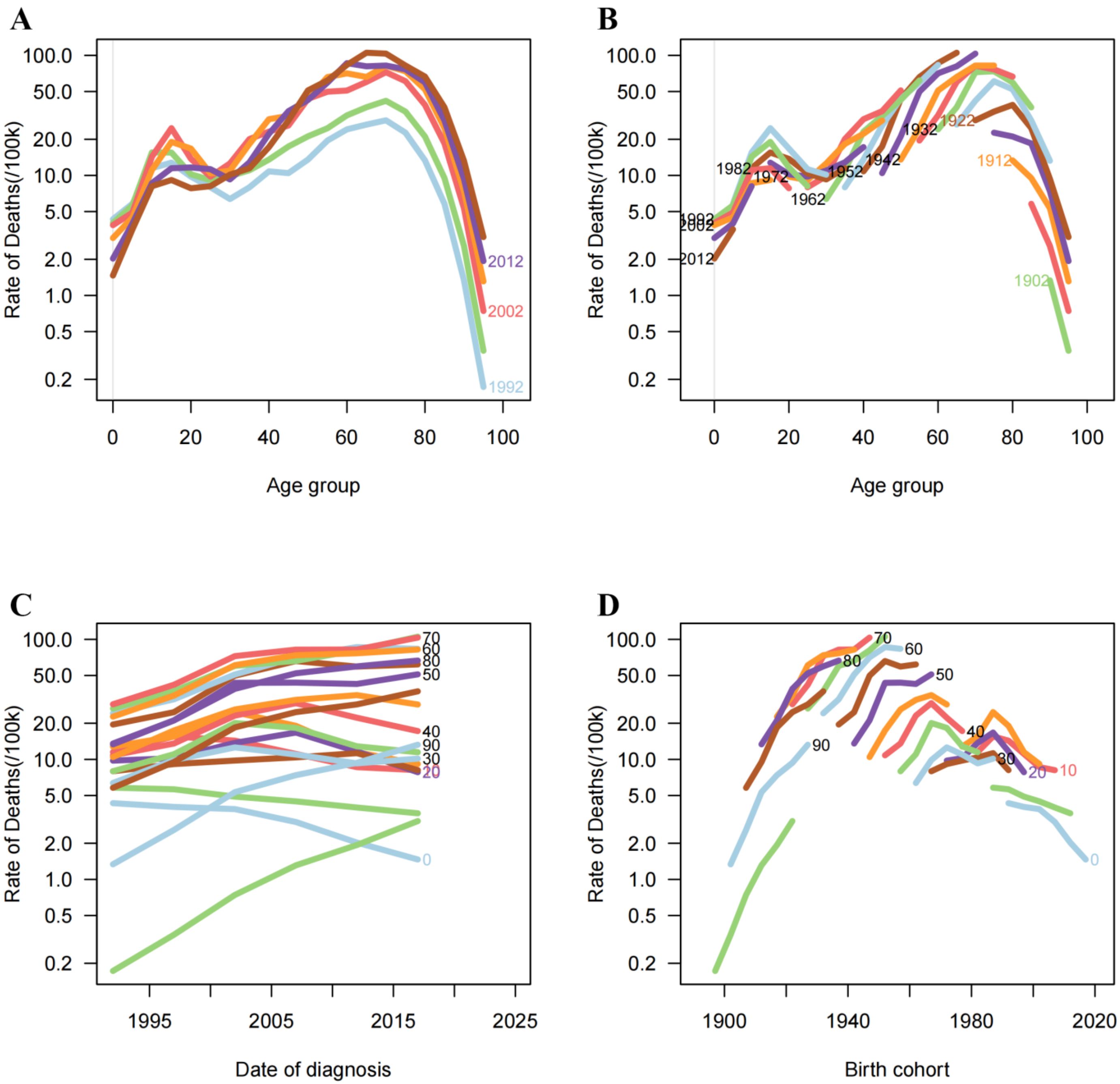
A
B
C
D
Rate of Deaths(/100k)
100.0
50.0
20.0
10.0
5.0
2.0
1.0
0.5
0.2
Age group
0
20
40
60
80
100
2012
2002
1992
1982
1972
1962
1952
1942
1932
1922
1912
1902
Date of diagnosis
1995
2005
2015
2025
70
60
80
50
40
90
30
20
0
Birth cohort
1900
1940
1980
2020
10

Supplementary Table 1. Incidence and Age-Standardized Rates of Malignant Neoplasms of Bone and Articular Cartilage in 1992 and 2021, Alongside Age-Specific Temporal Trends from 1992 to 2021.

Age	1992		2021		1992-2021
	Incident cases No.×10³ (95% UI)	ASR per 100,000 No. (95% UI)	Incident cases No.×10³ (95% UI)	ASR per 100,000 No. (95% UI)	EAPC No. (95% CI)
<5 years	278.299 (168.087-574.411)	0.254 (0.154-0.525)	133.563 (83.895-187.019)	0.172 (0.108-0.241)	-0.551 (-1.631-0.542)
10-14 years	520.672 (346.294-916.332)	0.521 (0.346-0.917)	820.348 (564.553-1055.664)	0.952 (0.655-1.225)	2.289 (1.752-2.828)
15-19 years	521.349 (338.878-945.732)	0.455 (0.296-0.825)	699.207 (469.064-910.124)	0.936 (0.628-1.219)	2.557 (1.99-3.127)
20-24 years	302.088 (185.165-566.64)	0.233 (0.143-0.438)	402.947 (250.244-554.494)	0.551 (0.342-0.758)	3.202 (2.674-3.732)
25-29 years	271.505 (163.362-534.019)	0.215 (0.129-0.423)	494.488 (297.206-679.015)	0.572 (0.344-0.785)	3.835 (3.297-4.377)
30-34 years	198.596 (117.037-386.25)	0.226 (0.133-0.44)	748.354 (458.884-1037.333)	0.618 (0.379-0.856)	3.902 (3.208-4.6)
35-39 years	298.356 (181.337-548.119)	0.312 (0.19-0.573)	844.137 (521.728-1150.672)	0.797 (0.492-1.086)	3.49 (2.725-4.26)
40-44 years	297.05 (177.011-564.366)	0.395 (0.235-0.75)	958.654 (582.976-1294.336)	1.047 (0.637-1.414)	3.711 (2.941-4.488)
45-49 years	250.215 (147.008-475.357)	0.445 (0.261-0.845)	1319.844 (779.966-1787.47)	1.196 (0.707-1.62)	3.995 (3.177-4.82)

5-9 years	426.197 (276.728-799.689)	0.379 (0.246-0.711)	626.755 (420.905-855.134)	0.654 (0.439-0.893)	2.179 (1.597-2.765)
50-54 years	327.588 (198.733-613.747)	0.685 (0.416-1.284)	2058.599 (1231.684-2845.533)	1.703 (1.019-2.354)	3.635 (2.796-4.481)
55-59 years	465.215 (280.696-875.693)	1.032 (0.623-1.943)	2682.511 (1619.097-3665.897)	2.44 (1.473-3.334)	3.598 (2.753-4.45)
60-64 years	520.127 (314.793-970.198)	1.399 (0.847-2.609)	2596.649 (1554.421-3571.661)	3.557 (2.129-4.892)	3.997 (3.223-4.776)
65-69 years	541.437 (330.897-989.766)	1.879 (1.148-3.434)	3563.953 (2066.487-4992.959)	4.646 (2.694-6.509)	3.728 (3.038-4.423)
70-74 years	524.048 (333.882-940.28)	2.621 (1.67-4.702)	3250.657 (1904.228-4463.157)	6.099 (3.573-8.374)	3.385 (2.684-4.092)
75-79 years	361.246 (226.442-645.288)	2.942 (1.844-5.256)	2304.255 (1390.132-3103.764)	6.958 (4.197-9.372)	3.476 (2.776-4.182)
80-84 years	179.454 (110.317-331.061)	3.124 (1.92-5.763)	1398.298 (841.86-1890.317)	7.065 (4.254-9.551)	3.325 (2.607-4.047)
85-89 years	68.613 (42.17-123.573)	3.525 (2.167-6.349)	764.226 (443.004-1057.755)	8.023 (4.651-11.104)	3.259 (2.485-4.039)
90-94 years	11.593 (7.216-21.323)	3.287 (2.046-6.047)	234.676 (137.8-320.795)	8.004 (4.7-10.941)	3.593 (2.766-4.427)
95+ years	1.016 (0.566-1.966)	2.318 (1.292-4.486)	35.686 (20.733-53.626)	5.584 (3.244-8.391)	3.549 (2.831-4.272)

ASR, age-standardized rate; CI, confidence interval; EAPC, estimated annual percentage change; UI, uncertainty interval.

Supplementary Table 2. Prevalence and Age-Standardized Rates of Malignant Neoplasms of Bone and Articular Cartilage in 1992 and 2021, Alongside Age-Specific Temporal Trends from 1992 to 2021.

Age	1992		2021		1992-2021
	Prevalent Cases No.×10^3 (95% UI)	ASR per 100,000 No. (95% UI)	Prevalent Cases No.×10^3 (95% UI)	ASR per 100,000 No. (95% UI)	EAPC No. (95% CI)
10-14 years	3658.265 (2434.538-6425.547)	3.66 (2.436-6.428)	5789.213 (3987.358-7450.669)	6.717 (4.626-8.644)	2.308 (1.771-2.849)
15-19 years	3737.841 (2430.103-6781.517)	3.261 (2.12-5.916)	5030.988 (3374.714-6547.794)	6.737 (4.519-8.769)	2.574 (2.005-3.145)
20-24 years	2102.782 (1290.287-3935.875)	1.624 (0.997-3.04)	2824.016 (1752.976-3885.64)	3.859 (2.396-5.31)	3.237 (2.71-3.767)
25-29 years	1867.963 (1124.59-3680.075)	1.48 (0.891-2.917)	3410.245 (2048.376-4686.428)	3.943 (2.369-5.419)	3.856 (3.314-4.401)
30-34 years	1388.879 (817.516-2704.336)	1.583 (0.932-3.082)	5271.44 (3228.216-7326.248)	4.351 (2.665-6.047)	3.941 (3.245-4.641)
35-39 years	2054.336 (1248.746-3772.882)	2.148 (1.306-3.945)	5842.79 (3611.014-7966.319)	5.514 (3.408-7.518)	3.517 (2.75-4.29)
40-44 years	2044.309 (1219.012-3882.283)	2.717 (1.62-5.159)	6648.155 (4043.936-8973.983)	7.263 (4.418-9.804)	3.746 (2.973-4.525)
45-49 years	1716.589 (1007.638-3262.317)	3.05 (1.791-5.797)	9158.367 (5404.335-12410.082)	8.302 (4.899-11.249)	4.042 (3.221-4.87)
5-9 years	2980.049 (1934.292-5592.294)	2.651 (1.721-4.974)	4396.227 (2952.348-5998.658)	4.59 (3.083-6.264)	2.195 (1.612-2.782)

50-54 years	2189.572 (1329.52-4104.257)	4.579 (2.781-8.583)	13977.455 (8356.727-19305.795)	11.565 (6.914-15.974)	3.696 (2.853-4.546)
55-59 years	3048.648 (1840.355-5734.109)	6.763 (4.083-12.72)	18006.141 (10866.254-24606.205)	16.378 (9.884-22.381)	3.687 (2.839-4.541)
60-64 years	3322.869 (2013.963-6205.105)	8.937 (5.417-16.689)	17153.393 (10268.811-23545.202)	23.496 (14.066-32.251)	4.112 (3.337-4.892)
65-69 years	3320.155 (2031.515-6060.19)	11.521 (7.049-21.029)	22867.277 (13251.677-32049.903)	29.813 (17.276-41.784)	3.886 (3.193-4.583)
70-74 years	3030.095 (1933.056-5424.042)	15.153 (9.667-27.125)	20086.962 (11821.526-27576.941)	37.689 (22.181-51.743)	3.638 (2.935-4.347)
75-79 years	1968.605 (1237.404-3506.601)	16.033 (10.078-28.559)	13809.286 (8306.309-18519.46)	41.696 (25.08-55.918)	3.834 (3.131-4.541)
80-84 years	832.539 (513.888-1530.244)	14.493 (8.946-26.64)	7366.624 (4437.875-9965.248)	37.22 (22.423-50.35)	3.784 (3.073-4.5)
85-89 years	250.195 (153.841-449.496)	12.855 (7.904-23.095)	3218.577 (1857.444-4446.912)	33.788 (19.499-46.683)	3.763 (3.008-4.525)
90-94 years	31.356 (19.674-57.894)	8.892 (5.579-16.418)	761.371 (455.322-1050.082)	25.968 (15.529-35.815)	4.267 (3.441-5.099)
95+ years	0.747 (0.415-1.431)	1.705 (0.947-3.266)	31.596 (18.362-47.897)	4.944 (2.873-7.494)	4.291 (3.578-5.009)

ASR, age-standardized rate; CI, confidence interval; EAPC, estimated annual percentage change; UI, uncertainty interval.

Supplementary Table 3. Mortality and Age-Standardized Rates of Malignant Neoplasms of Bone and Articular Cartilage in 1992 and 2021, Alongside Age-Specific Temporal Trends from 1992 to 2021.

Age	1992		2021		1992-2021
	Death cases No.×10^3 (95% UI)	ASR per 100,000 No. (95% UI)	Death cases No.×10^3 (95% UI)	ASR per 100,000 No. (95% UI)	EAPC No. (95% CI)
10-14 years	220.706 (147.238-402.792)	0.221 (0.147-0.403)	213.171 (148.754-269.39)	0.247 (0.173-0.313)	0.412 (-0.108-0.936)
15-19 years	361.303 (235.648-662.922)	0.315 (0.206-0.578)	295.25 (199.86-386.498)	0.395 (0.268-0.518)	0.654 (0.107-1.205)
20-24 years	268.933 (164.748-501.095)	0.208 (0.127-0.387)	235.199 (150.794-320.039)	0.321 (0.206-0.437)	1.462 (0.918-2.009)
25-29 years	181.349 (109.651-354.597)	0.144 (0.087-0.281)	206.736 (126.925-277.769)	0.239 (0.147-0.321)	1.916 (1.386-2.449)
30-34 years	119.958 (70.709-235.386)	0.137 (0.081-0.268)	281.818 (174.959-385.871)	0.233 (0.144-0.318)	1.967 (1.271-2.669)
35-39 years	191.05 (117.207-356.767)	0.2 (0.123-0.373)	329.505 (210.387-443.505)	0.311 (0.199-0.419)	1.558 (0.801-2.321)
40-44 years	224.059 (133.107-428.352)	0.298 (0.177-0.569)	442.872 (276.789-591.781)	0.484 (0.302-0.647)	1.802 (1.059-2.551)
45-49 years	213.995 (125.291-413.988)	0.38 (0.223-0.736)	707.882 (433.192-963.259)	0.642 (0.393-0.873)	2.121 (1.341-2.906)
5-9 years	120.632 (78.295-223.582)	0.107 (0.07-0.199)	108.529 (73.101-143.449)	0.113 (0.076-0.15)	0.262 (-0.309-0.837)

50-54 years	304.896 (184.121-569.465)	0.638 (0.385-1.191)	1237.399 (761.905-1675.254)	1.024 (0.63-1.386)	1.838 (1.012-2.671)
55-59 years	459.509 (279.59-864.748)	1.019 (0.62-1.918)	1776.392 (1109.563-2444.437)	1.616 (1.009-2.223)	1.91 (1.055-2.771)
60-64 years	533.31 (323.046-982.708)	1.434 (0.869-2.643)	1815.405 (1113.383-2487.88)	2.487 (1.525-3.408)	2.383 (1.579-3.193)
65-69 years	575.806 (352.513-1051.677)	1.998 (1.223-3.649)	2727.168 (1585.018-3750.169)	3.555 (2.066-4.889)	2.343 (1.585-3.107)
70-74 years	586.226 (373.684-1053.198)	2.932 (1.869-5.267)	2783.217 (1684.699-3770.251)	5.222 (3.161-7.074)	2.287 (1.501-3.079)
75-79 years	432.556 (271.879-775.579)	3.523 (2.214-6.317)	2168.54 (1334.738-2897.87)	6.548 (4.03-8.75)	2.491 (1.709-3.279)
80-84 years	228.218 (141.801-421.805)	3.973 (2.469-7.343)	1521.823 (932.213-2033.817)	7.689 (4.71-10.276)	2.708 (1.914-3.509)
85-89 years	94.57 (58.233-170.405)	4.859 (2.992-8.755)	899.606 (539.168-1240.772)	9.444 (5.66-13.025)	2.649 (1.795-3.511)
90-94 years	17.672 (11.047-32.775)	5.011 (3.133-9.294)	261.859 (160.565-349.123)	8.931 (5.476-11.907)	2.273 (1.389-3.165)
95+ years	1.759 (0.987-3.403)	4.012 (2.25-7.763)	49.163 (28.975-68.682)	7.693 (4.534-10.747)	2.639 (1.85-3.434)

ASR, age-standardized rate; CI, confidence interval; EAPC, estimated annual percentage change; UI, uncertainty interval.

Supplementary Table 4. DALYs and Age-Standardized Rates of Malignant Neoplasms of Bone and Articular Cartilage in 1992 and 2021, Alongside Age-Specific Temporal Trends from 1992 to 2021.

Age	1992		2021		1992-2021
	DALYs cases No.×10^3 (95% UI)	ASR per 100,000 No. (95% UI)	DALYs cases No.×10^3 (95% UI)	ASR per 100,000 No. (95% UI)	EAPC No. (95% CI)
10-14 years	17473.596 (11649.276-31778.308)	17.481 (11.654-31.792)	17089.232 (11933.271-21648.617)	19.827 (13.845-25.116)	0.463 (-0.055-0.983)
15-19 years	26581.102 (17317.369-48615.485)	23.19 (15.108-42.412)	21934.081 (14802.187-28738.195)	29.374 (19.823-38.486)	0.684 (0.136-1.236)
20-24 years	18412.036 (11297.971-34345.339)	14.221 (8.726-26.527)	16182.675 (10368.511-22045.238)	22.115 (14.17-30.127)	1.485 (0.936-2.037)
25-29 years	11551.782 (6999.268-22628.845)	9.156 (5.547-17.935)	13254.026 (8144.99-17790.235)	15.326 (9.418-20.571)	1.96 (1.426-2.496)
30-34 years	7058.866 (4154.986-13859.256)	8.045 (4.735-15.795)	16746.631 (10389.082-22943.601)	13.823 (8.575-18.938)	2.025 (1.331-2.724)
35-39 years	10267.459 (6316.117-19183.505)	10.737 (6.605-20.061)	17990.759 (11448.707-24294.387)	16.978 (10.804-22.927)	1.614 (0.858-2.377)
40-44 years	10947.195 (6499.783-20960.691)	14.548 (8.638-27.855)	21842.478 (13548.155-29182.061)	23.863 (14.801-31.881)	1.839 (1.09-2.593)

45-49 years	9382.345 (5487.598-18144.881)	16.672 (9.751-32.243)	31243.859 (19115.515-42331.157)	28.321 (17.327-38.371)	2.16 (1.38-2.946)
5-9 years	10289.488 (6673.841-19137.637)	9.153 (5.936-17.023)	9421.96 (6327.816-12400.426)	9.838 (6.607-12.948)	0.337 (-0.237-0.914)
50-54 years	11853.62 (7152.982-22141.066)	24.79 (14.959-46.305)	48557.035 (29788.129-66128.94)	40.177 (24.647-54.716)	1.88 (1.055-2.711)
55-59 years	15716.034 (9531.264-29542.056)	34.863 (21.143-65.534)	61433.37 (38477.956-84775.528)	55.878 (34.998-77.109)	1.952 (1.097-2.814)
60-64 years	15753.313 (9550.923-29096.071)	42.369 (25.687-78.255)	53975.931 (32935.752-73774.383)	73.934 (45.114-101.054)	2.415 (1.608-3.229)
65-69 years	14361.825 (8795.043-26237.739)	49.835 (30.519-91.045)	68566.34 (40084.069-94676.368)	89.391 (52.258-123.432)	2.389 (1.637-3.147)
70-74 years	12124.413 (7725.499-21784.981)	60.632 (38.634-108.943)	58011.268 (35003.086-78399.486)	108.846 (65.676-147.101)	2.318 (1.539-3.102)
75-79 years	7237.429 (4529.474-13001.437)	58.945 (36.89-105.89)	36469.544 (22423.358-48668.553)	110.117 (67.706-146.951)	2.503 (1.725-3.287)
80-84 years	3025.789 (1868.67-5588.892)	52.675 (32.531-97.296)	20112.654 (12269.7-26978.601)	101.621 (61.994-136.312)	2.7 (1.911-3.494)
85-89 years	1000.033 (615.591-1801.382)	51.382 (31.629-92.555)	9508.154 (5664.461-12957.12)	99.815 (59.465-136.022)	2.65 (1.808-3.499)

90-94 years	161.632 (101.018-297.694)	45.836 (28.647-84.421)	2429.602 (1478.478-3206.849)	82.865 (50.426-109.375)	2.328 (1.454-3.21)
95+ years	14.831 (8.276-28.727)	33.833 (18.88-65.532)	414.68 (243.701-582.71)	64.885 (38.132-91.177)	2.649 (1.859-3.446)

ASR, age-standardized rate; CI, confidence interval; EAPC, estimated annual percentage change; UI, uncertainty interval.

Supplementary Table 5. Projection of ASR (per 100,000) for Incidence, Prevalence, Mortality, and DALYs of MNBAC in China Over the Next Decade.

Measures	2022	2023	2024	2025	2026	2027	2028	2029	2030	2031
Incidence										
Both	1.40(1.38,1.42)	1.38(1.31,1.45)	1.37(1.23,1.50)	1.35(1.13,1.57)	1.34(1.03,1.65)	1.32(0.91,1.73)	1.31(0.79,1.82)	1.29(0.66,1.92)	1.27(0.52,2.02)	1.26(0.38,2.13)
Male	1.76(1.73,1.78)	1.73(1.67,1.80)	1.71(1.58,1.84)	1.69(1.47,1.90)	1.66(1.34,1.98)	1.64(1.20,2.07)	1.61(1.04,2.18)	1.58(0.86,2.31)	1.56(0.67,2.44)	1.53(0.47,2.59)
Female	1.05(1.02,1.08)	1.03(0.94,1.11)	1.00(0.84,1.17)	0.98(0.71,1.24)	0.95(0.58,1.33)	0.93(0.43,1.43)	0.91(0.27,1.54)	0.88(0.10,1.67)	0.86(-0.09,1.80)	0.83(-0.28,1.94)
Prevalence										
Both	9.05(8.91,9.19)	8.96(8.54,9.37)	8.87(8.04,9.71)	8.79(7.45,10.14)	8.71(6.78,10.65)	8.63(6.05,11.21)	8.55(5.28,11.81)	8.46(4.46,12.46)	8.38(3.60,13.16)	8.29(2.69,13.89)

Male	11.17(11.03,11.31)	11.05(10.65,11.44)	10.92(10.14,11.70)	10.79(9.50,12.08)	10.66(8.75,12.57)	10.52(7.88,13.16)	10.39(6.93,13.85)	10.25(5.87,14.63)	10.12(4.74,15.50)	9.98(3.52,16.44)
Female	6.85(6.68,7.02)	6.69(6.18,7.19)	6.52(5.51,7.54)	6.36(4.72,8.00)	6.20(3.84,8.56)	6.03(2.87,9.20)	5.87(1.83,9.92)	5.71(0.72,10.70)	5.55(-0.46,11.55)	5.38(-1.69,12.46)
Deaths										
Both	0.91(0.88,0.93)	0.88(0.81,0.95)	0.86(0.72,1.00)	0.84(0.61,1.07)	0.82(0.49,1.14)	0.80(0.36,1.23)	0.77(0.23,1.32)	0.75(0.08,1.43)	0.73(-0.08,1.54)	0.71(-0.25,1.66)
Male	1.15(1.12,1.18)	1.13(1.05,1.20)	1.10(0.96,1.25)	1.08(0.84,1.31)	1.05(0.72,1.39)	1.03(0.57,1.48)	1.00(0.42,1.59)	0.98(0.25,1.71)	0.95(0.07,1.84)	0.93(-0.11,1.97)
Female	0.69(0.66,0.71)	0.67(0.59,0.74)	0.65(0.52,0.78)	0.63(0.43,0.83)	0.61(0.33,0.88)	0.58(0.22,0.95)	0.56(0.11,1.02)	0.54(-0.01,1.10)	0.52(-0.14,1.19)	0.50(-0.27,1.28)
DALYs										
Both	29.10(28.38,29.83)	28.72(26.54,30.90)	28.33(24.12,32.55)	27.93(21.32,34.54)	27.51(18.27,36.75)	27.09(15.05,39.13)	26.67(11.67,41.68)	26.26(8.10,44.41)	25.84(4.35,47.33)	25.43(0.41,50.44)
Male	36.43(35.63,37.23)	36.03(33.80,38.27)	35.66(31.37,39.95)	35.30(28.40,42.19)	34.94(24.94,44.94)	34.59(21.03,48.15)	34.24(16.72,51.76)	33.89(12.03,55.74)	33.54(7.01,60.07)	33.19(1.67,64.72)
Female	21.60(20.77,22.42)	21.06(18.68,23.43)	20.52(15.75,25.28)	19.98(12.30,27.65)	19.44(8.42,30.46)	18.90(4.15,33.64)	18.36(-0.46,37.18)	17.82(-5.39,41.03)	17.28(-10.62,45.17)	16.74(-16.12,49.60)

ASR, Age-Standardized Rates; DALYs, Disability-Adjusted Life Years; MNBAC, Malignant Neoplasms of Bone and Articular Cartilage

Supplementary Table 6: ARIMA model parameters along with their respective AIC and BIC values for predicting the ASR (per 100,000) of all four metrics for MNBAC over the next 10 years in China.

**Measures**			
Incidence	Parameters	AIC	BIC
Both	ARIMA(2,2,0)	-166.53	-162.53
Male	ARIMA(1,2,0)	-164.74	-162.08
Female	ARIMA(0,2,2)	-152.39	-148.39
Prevalence			
Both	ARIMA(2,2,0)	-65.09	-61.10
Male	ARIMA(1,2,0)	-65.57	-62.90
Female	ARIMA(0,2,2)	-51.51	-47.51
Deaths			
Both	ARIMA(0,2,2)	-163.744	-159.7474
Male	ARIMA(1,2,0)	-155.0548	-152.3904
Female	ARIMA(0,2,1)	-159.4624	-156.798
DALYs			
Both	ARIMA(2,2,0)	28.38768	32.38429
Male	ARIMA(1,2,0)	32.91777	35.58218
Female	ARIMA(0,2,2)	35.72268	39.7193

ARIMA, Autoregressive Integrated Moving Average; AIC, Akaike Information Criterion; BIC, Bayesian Information Criterion; ASR, Age-Standardized Rates; MNBAC, malignant neoplasms of bone and articular cartilage
